# Capillary Effect Enhancement in a Plastic Capillary Tube by Nanostructured Surface

**DOI:** 10.3390/polym13040628

**Published:** 2021-02-19

**Authors:** Kazuma Kurihara, Ryohei Hokari, Naoki Takada

**Affiliations:** 1 Advanced Manufacturing Research Institute, National Institute of Advanced Industrial Science and Technology, AIST, 1-2-1 Namiki, Tsukuba, Ibaraki 305–8564, Japan; hokari.ryohei@aist.go.jp; 2 Research Institute for Energy Conservation, National Institute of Advanced Industrial Science and Technology, AIST, 1-2-1 Namiki, Tsukuba, Ibaraki 305–8564, Japan; naoki-takada@aist.go.jp

**Keywords:** wettability control, injection moulding process, nanostructure, hydrophilic surface, capillary effect

## Abstract

We investigated the enhancement of the capillary effect in a plastic capillary tube using only a nanostructured surface. Since plastic is a hydrophobic material, the capillary effect does not emerge without an additional coating or plasma treatment process. Therefore, capillary effect enhancement by the nanostructure fabrication method is expected to reduce the cost and minimise the contamination produced in the human body. By combining a hydrophilic nylon resin and a nanostructure at the tip of the plastic pipette, we could confirm that the capillary effect was produced solely by the tube fabrication process. The produced capillary effect increased linearly with increasing nanostructure height when a standard solution with a surface tension of 70 mN·m^−1^ was used. Thus, we can conclude that including the plastic part with nanostructure can be useful for biomedical applications. In addition, we suggest that the proposed method is highly effective in controlling the wetting properties of plastic surfaces, compared to the typical coating or plasma treatment processes.

## 1. Introduction

The surface wettability control of plastic materials is very important for developing plastic-based medical instruments, food packages, optical displays, and a wide variety of industrial devices [1,2,3,4,5]. Surface wettability control has been achieved via plasma treatment and chemical or physical surface coatings, which have been widely adapted for various types of materials, including polymers. The chemical or physical surface coating methods, in which a material with different wetting properties is coated on the surface, allow good control of surface wettability in plastics [6,7]. However, the applications of these methods are limited because of the peeling of the additional coated layer, which severely impedes their medical and biological applications. To mitigate these drawbacks, an alternative method that does not involve an additional coating process is a necessary requirement for controlling the wetting property of material surfaces. The plasma treatment method is one of the most promising methods to realize surface wettability control without an additional coating process [8,9]. This method produces OH radicals on the material surface, whose hydrophilic properties depend on the surface density of the OH radicals [10]. However, the hydrophilicity of the surface cannot be maintained for a long time because the number of OH radicals decreases over time. In particular, retaining the surface hydrophilicity becomes a challenging task in the case of hydrophobic polymers. Therefore, the selection of plastic-based materials is important for real-world applications because surface wettability depends strongly on the material property. To improve the wetting property of polymers, additive compounds have also been used [11,12,13]. These compounds, when mixed with a polymer, allow the gradual appearance of hydrophilicity on the polymer surface. The wetting property can be controlled by this method via composite material technology. Although the hydrophilic properties of polymers can be maintained for a longer duration by selecting appropriate materials and increasing the additive compound concentration, the retained hydrophilicity is insufficient for the aforementioned applications of plastics. Therefore, it is difficult to produce the capillary effect by material selection and increasing the additive compound concentration.

To enhance the wetting property, several authors have reported that the micro- or nanostructures on the material surface can be used to control the surface wettability and sliding angle [14,15,16,17,18]. The wettability of a surface structure can be described by the Cassie–Baxter model or the Wenzel model [19,20]. According to the Wenzel model, the hydrophilicity of a surface (<90°) increases with increasing surface roughness. The surface roughness is increased by the presence of surface micro- or nanostructures, which in turn govern the hydrophilic properties of the surface. In addition, when the pitch of the structures is less than half of the visible wavelength (i.e., around 250 nm), the substrate becomes transparent due to the subwavelength structure effect, because the incident light is not diffracted in this case [21,22,23]. In polymers, these surface structures are fabricated via the structure transfer processes, such as ultraviolet nanoimprinting, hot embossing, or injection moulding [24,25,26,27,28,29], which allow us to realize wetting control at low costs [30,31,32]. Therefore, wetting control by nanostructures is an effective method for medical and food packaging applications because plastic components that are used in the human body cannot be subjected to physical or chemical coatings, owing to contamination hazards. Thus, nanostructures are advantageous in improving the wetting properties and decreasing the contamination. 

Although, theoretically, the nanostructures are strongly expected to control the wetting property, the resulting hydrophilic property enhancement is limited because the theoretical description of the structural shape and surface roughness are based on the Wenzel model and the Cassie–Baxter model [19,20]. In addition, the wetting property investigations using micro- or nanostructures have been reported only for 2D surfaces because the fabrication process is difficult; this is due to the limitations of the nanostructure transfer process. Further, the availability of suitable plastic materials, which determines the wetting property, is limited because of the hydrophobicity of plastic. Therefore, although structure-induced wetting enhancement is strongly expected, especially in medical capillary tubes because of the reduced contamination, the possibility of enhancing the capillary effect via surface structures has not yet been well investigated, owing to the fabrication difficulty with a 3D shape. Thus, in this study, we investigated the influence of surface nanostructures on the capillary effect of a plastic capillary tube. In addition, we investigated the feasibility of enhancing the capillary effect of the plastic tube only by the injection moulding process, without additional coating or plasma treatment. Based on this investigation, in this paper, we discuss the possibility of using a capillary tube as a medical testing equipment.

## 2. Design and Numerical Simulation of the Capillary Tube

A nanostructure is expected to produce the capillary effect in a capillary tube because it influences the wetting property, thereby changing the capillary pressure [33]. The capillary tube with the nanostructure should be fabricated via the injection moulding technique. However, in this technique, the nanostructure cannot be added on the side wall of the capillary tube because of the moulding direction. Based on the fabrication limitation, the effect of the capillary tube must be carefully estimated in terms of the differences in the surface wetting properties. 

To qualitatively investigate the effect of the nanostructured surface on the capillary tube, a simple numerical simulation of the two-phase fluid flow was performed in 2D by using the phase-field, model-based computational fluid dynamics (CFD) method [34]. In the CFD method, an interface between different fluid phases is autonomously formed as a finite volumetric zone, across which physical properties vary continuously from one phase to another phase based on the free-energy theory [35]. As a result, conventional algorithms for the advection and reconstruction of interfaces are not necessarily required. The contact angle of a fluid phase on a solid surface can be set as a parameter of the surface wettability through a simple boundary condition at each position of the solid surface. Therefore, the main advantage of the CFD method over others is the efficient simulation of the motions of multiple interfaces that are attached to solid bodies with edges and heterogeneously wetted surfaces.

**Figure 1:**
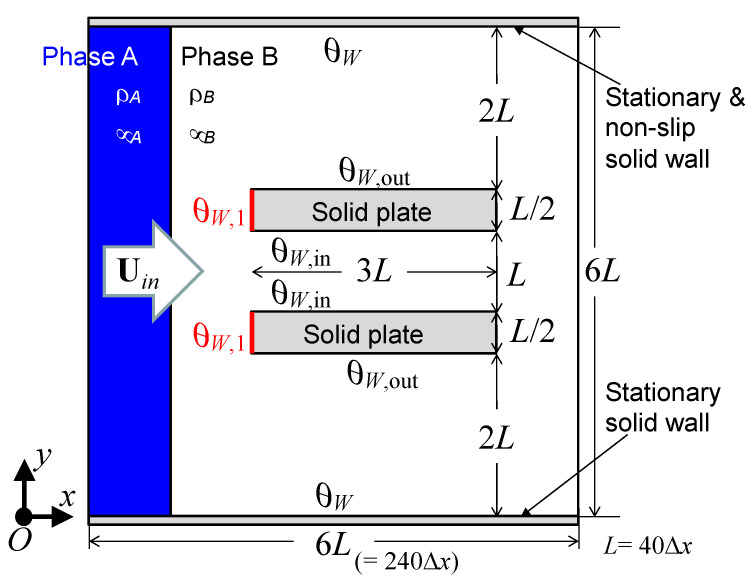
Illustration of the capillary tube model.

Figure 1 illustrates the calculation model. The computational domain is surrounded by uniform inflow and continuous outflow boundaries on the left and right, in the x-direction, and by stationary flat solid wall boundaries on the top and bottom, in the y-direction. The domain is uniformly divided into 240 × 240 square cells on a structured grid having a spatial resolution Δ*x* = Δ*y* = 1.25 × 10^−5^ m. 

For simplicity, the densities, *ρ_A_* and *ρ_B_*, of the two fluid phases, A and B, respectively, are defined as 998.2 kg·m^−3^, and the viscosities, *μ_A_* and *μ_B_*, are defined as 1.0 × 10^−3^ Pa·s. The interfacial tension, *σ*, between phases A and B is defined as 7 × 10^−2^ N·m^−1^. The two stationary and parallel solid plates shown in Figure 1 illustrate a simplified cross-sectional model of the capillary tube. The distance, *L*, between the plates is 40 Δ*x*, which is equivalent to a 500 μm inner diameter of the capillary tube. The thickness and length of the plates are 250 μm and 1.5 mm, respectively. *θ_W_,_in_* and *θ_W,out_* represent the inner and outer surface contact angles of the plates, respectively. In addition, the contact angle, *θ_W,1_*, is set at the front faces of the plates, which form the tip of the capillary tube. Here, it is assumed that the wetting property at the front faces can be varied by the nanostructure-induced surface roughness owing to the structure effect, according to the Cassie–Baxter or Wenzel theory. In the simulation, the fluid phase A flows at a constant volumetric flow rate with a uniform velocity, *U_in_*, in the x-direction. The dimensionless characteristic parameters (i.e., the Ohnesorge number (*Oh*), Reynolds number (*Re*), and capillary number (*Ca*)) are set to *Oh* = *μ*_A_/(*ρ*_A_*σL*)^0.5^ = 5.35 × 10^−3^, *Re* = *ρ*_A_*LU_in_* /*μ*_A_ = 0.9982, and *Ca* = *μ*_A_*U_in_* /*σ* = 2.857 × 10^−5^, respectively. 

**Figure 2:**
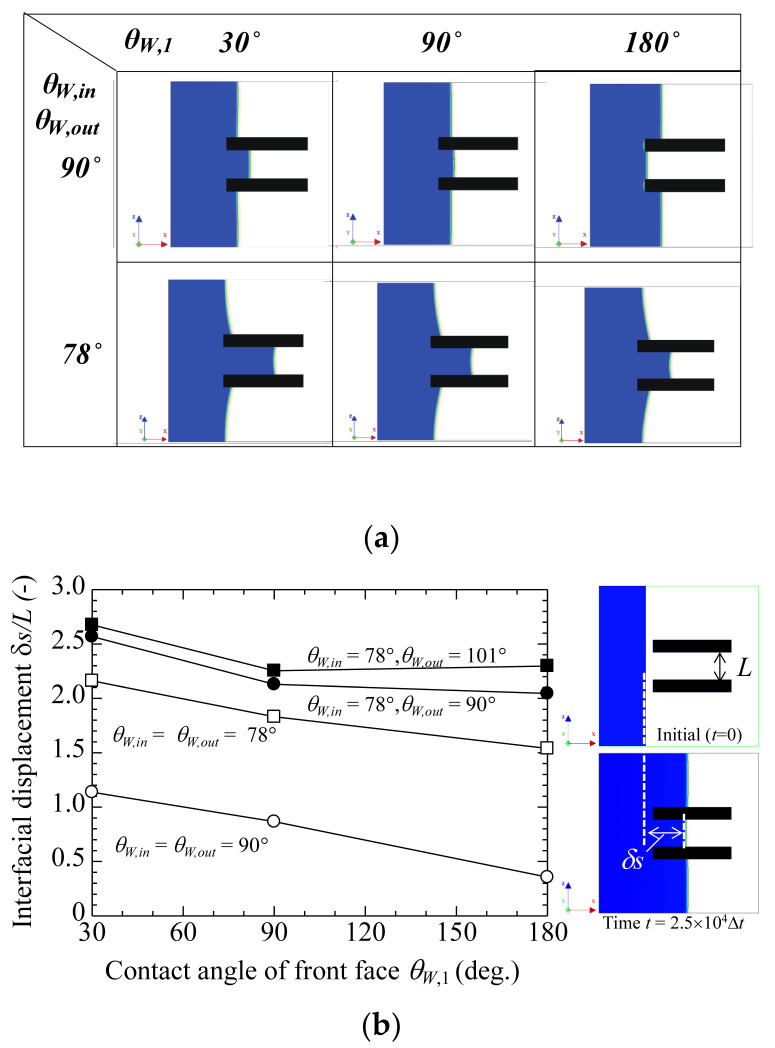
Penetration effect due to different contact angles, *θ_W,out_*, *θ_W,in_*, and *θ_W,1_*. (**a**) Snapshots for time, *t* = 25,000 ∆t. (**b**) Interfacial displacement, *∂s/L*, as a function of contact angle of front face, *θ_W,1_*.

Figure 2 shows the numerical results of the effect of *θ_W,in_*, *θ_W,out_*, and *θ_W,1_* on the penetration of phase A into the gap between the plates. The images shown in Figure 2a are snapshots of the two-phase spatial distribution at time, *t* = 25,000 *Δt*, where *Δt* is the discrete time increase. The capillary effect is increased by decreasing *θ_W,in_* and *θ_W,out_*, as shown in Figure 2b for the displacement of the two-phase interface from its initial position. According to the capillary action equation, which is expressed as *h = 2σcosθ/rσg,* the capillary effect depends on the contact angle θ between the liquid phase and solid surface. In this equation, *h*, *σ*, *r, ρ*, and *g* are the height of the liquid-phase-penetrating vertical capillary tube, surface tension of the liquid phase, radius of the capillary tube, density of the liquid phase, and acceleration due to gravity, respectively. The increase in the displacement with a decrease in *θ_W,__in_* is in accordance with that of a gas–liquid interface between parallel plates reported in a previous 3D simulation [36]. We consider that at smaller contact angles, the solid surfaces become more wettable to phase A to accelerate the spreading motion on the surfaces, and consequently increase the penetration of phase A into the gap. The capillary effect is strongly dependent on the wetting property of the base solid material. Surprisingly, the interfacial displacement, *∂s/L,* is increased by decreasing *θ_W,1_* because the wetting property of the surface at the tip, with *θ_W,1_*, becomes more hydrophilic. In this way, the capillary effect is also increased by the hydrophilic tip. 

As a result of the change in the surface wettability, we can consider that the effect observed at the tip of the capillary tube depends on trapped air bubbles. Thus, it is possible to enhance the capillary effect by increasing the hydrophilicity at only the tip of the capillary tube, and this hydrophilicity can be controlled only with surface nanostructures that are grown via the moulding process.

In the simulation, the interfacial tension was assumed to be equivalent to that of an actual two-phase system. For constant contact angles, a decrease in the interfacial tension reduces the penetration of phase A into the gap, thereby reducing the capillary effect on the fluid motion due to a decreased capillary pressure. If the inflow velocity, *U_in_*, is increased, the penetration speed of phase A decreases relative to that of the phase flowing outside the solid plates. This occurs because of the increase in the flow resistance, which acts on the phase at the front and inside faces of the plates. In our case, we assumed that the effect of surface nanostructure on the wettability was implicitly included in the contact angle as a macroscopic parameter of the surface wettability. The effect of an electric charge on the capillary pressure was not evaluated in the simulations because we focused on the two-phase fluid dynamics, without the charge effect, for simplicity.

## 3. Methods and Materials

**Figure 3:**
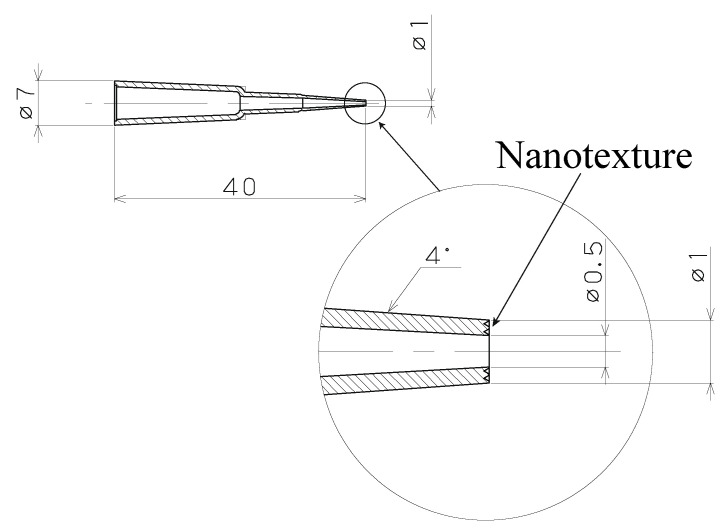
Schematic of the plastic pipette with the nanotexture.

Figure 3 shows a schematic of the plastic capillary tube with a nanostructure at the tip. The inner and outer diameters of the tip were 0.5 mm and 1 mm, respectively. The wall angle of the capillary tube was 4° for easy removal after the plastic-forming process. 

**Figure 4:**
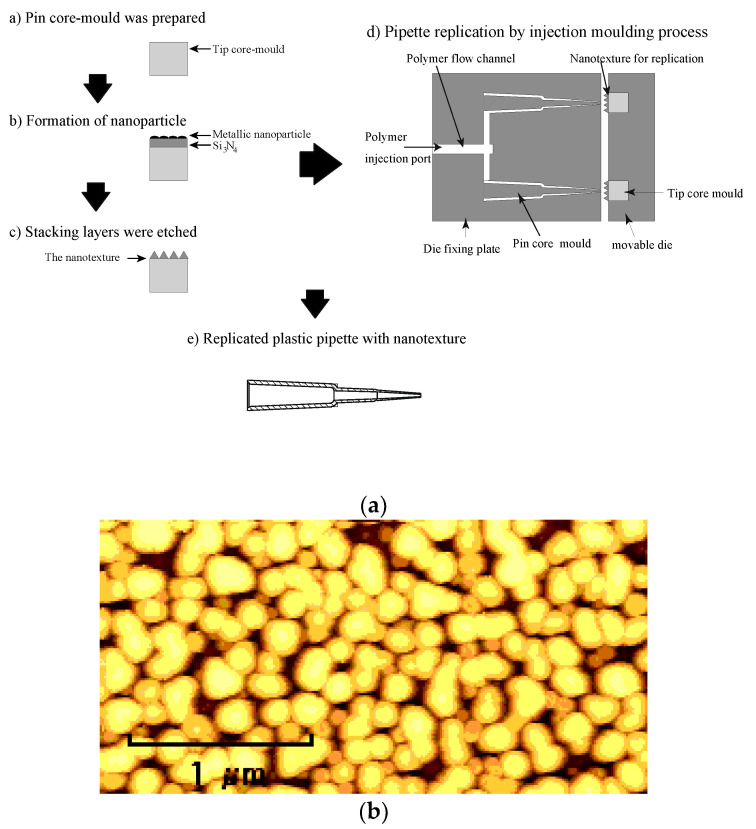
Illustration of the fabrication process of the nanostructured plastic capillary tube by injection moulding. (**a**) Fabrication process of the plastic pipette with nanotexture and (**b**) AFM image of the fabricated nanotexture.

The nanostructure was fabricated on the surface of a core mould to form the capillary tip. In the nanostructure fabrication process, first, we prepared the core mould. Next, stacked layers of Si_3_N_4_ and platinum oxide were deposited, with thicknesses of 300 nm and 8 nm, respectively, on the core mould via radiofrequency (RF) magnetron sputtering [25]. After the deposition of the stacked layers, the core mould was annealed and metallic nanoparticles were formed, after which the core mould was etched by reactive ion etching, followed by the fabrication of a high-aspect-ratio nanostructure on its the surface. Figure 4b shows an atomic force microscopy (AFM) image of the nanostructure fabricated on the mould. The diameter and pitch of the nanostructure were 150 nm and 200 nm, respectively, to maintain the transparency of the plastic tube. In addition, the mould height of the nanostructure was 230 nm. The mould was fabricated via dry processes only in order to produce curved moulds more easily. After the formation of the nanostructure, the melted polymer was injected into the mould, thereby filling the space for the capillary tube and simultaneously replicating the nanostructure. The nylon polymer ESA-9160N (produced by RIKEN TECHNOS Corp., Tokyo, Japan) was used for replicating the capillary tube.

A commercial contact angle meter (DM-300, Kyowa International Science Co., Ltd. Saitama, Japan) was used to study the water droplets. The volume of the water was fixed at 1 µL. The contact angles were measured at ten different points for each sample, to obtain an average value. The contact angles were then evaluated by the curve-fitting procedure. To evaluate the water wettability of the plastic plate with respect to the height of the nanostructure, we measured the ratio of the area with water and that without water. The evaluation area was 35 mm × 35 mm. A water sprayer was used to wet the surface. During the experiment, the atmospheric temperature and humidity were 23 ± 3 °C and 45% ± 10%, respectively. The replicated plate was set perpendicular to the ground level to eliminate the excessive water. In the capillary effect investigation, the tip of the pipette was carefully connected to a water droplet. A standard solution with a surface tension of 70 mN·m^−1^ was used in the experiment. The capillary effect was investigated by weight variation, which was measured by an electronic balance.

## 4. Results and Discussion

The hydrophilic properties of the outer and inner surfaces of plastic are very important for enhancing the capillary effect, as shown in Figure 2b. Therefore, the selection of suitable polymer-based materials is crucial for realizing wettability control. Table 1 shows the water contact angle for several types typical of plastic materials. The contact angle of the plastic plate was in the range of 74.5°–101.2°.

**Figure 5:**
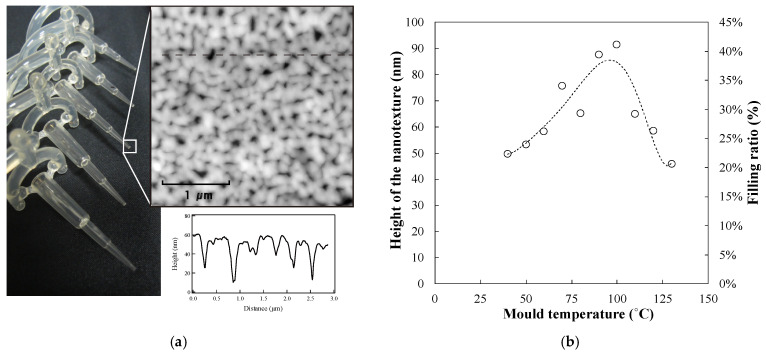
(**a**) AFM image of the replicated plastic tip surface and (**b**) replication height of the nanotexture as a function of the mould temperature.

The wetting property was drastically varied by the selection of plastic material. Since plastic is inherently hydrophobic, almost all plastic-based materials show higher hydrophobicity as compared to those coated by inorganic materials such as silicon oxide, with a contact angle of approximately 10°. This is the main difficulty in obtaining the capillary effect in plastic materials without coating or plasma treatment technology. Cyclo olefin polymer (COP), low-density polycarbonate resin (PC), and polypropylene resin (PP) show high hydrophobicity, whereas nylon resin (NY) and acrylic resin (PMMA) show hydrophilicity. In our experiment, the contact angle was measured at approximately 75°. In addition, the hydrophobic trend was enhanced by the nanostructure, according to the Wenzel theory. Thus, by combining the nanostructure with the aforementioned hydrophilic plastic materials (e.g., NY or PMMA), there is a possibility of producing the capillary effect of the plastic capillary tube without additional coating or plasma treatment technology. Figure 5a shows a photo and the corresponding AFM image of the plastic capillary tube with the nanostructure at the tip surface. Figure 5b shows the variations in the height of the nanostructures, with changes in the mould temperature. The filling ratio is estimated from the height and depth of the nanostructure on the mould and the replicated plate, respectively.

**Figure 6:**
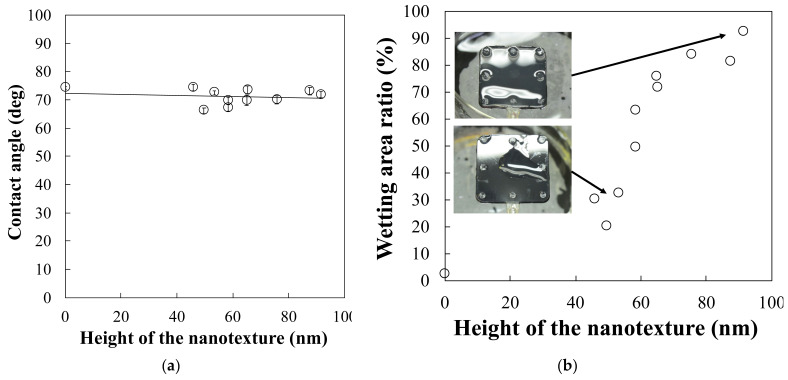
(**a**) Contact angle as a function of the height of the nanotexture and (**b**) wetting behaviour using the water sprayer.

The nanostructure height increases gradually when the mould temperature is less than 100 °C. The height of the nanostructure increases from 40 nm to 100 nm, and the filling ratio increases from 22% to 40%. According to the Andrade equation or the Williams–Landel–Ferry (WLF) equation, as NY starts melting at 105 °C, when the temperature of the polymer is less than the melting point, the viscosity of the polymer gradually decreases with increasing temperature [37,38]. Thus, the height of the replicated nanostructure is related to the mould temperature. In contrast, when the mould temperature is over 100 °C, the height of the nanostructure decreases rapidly because the melted polymer cannot be solidified at such high temperatures. The viscosity of the polymer is partially determined by the temperature of the mould in the injection moulding process. Thus, we can conclude that a capillary tube with a nanostructure can be fabricated by controlling the mould temperature. Figure 6 shows the wetting property as a function of the height of the nanostructure.

**Figure 7:**
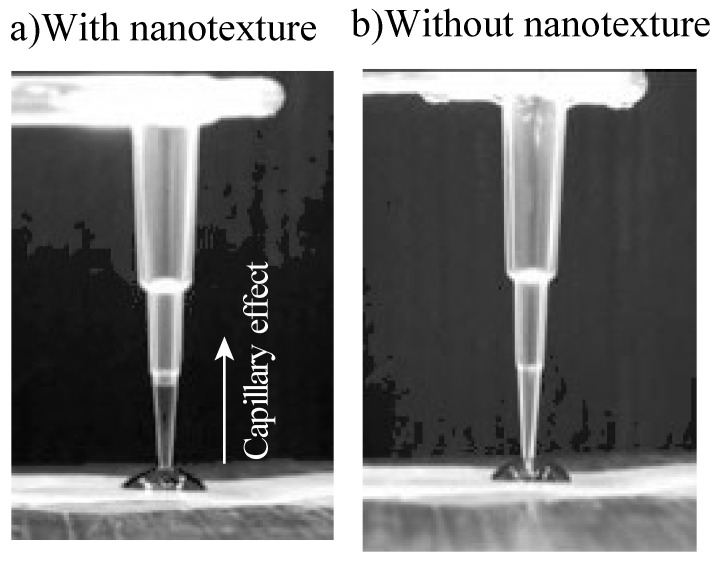
(**a**) Photographs of the replicated plastic pipettes with the nanotexture at the tip and (**b**) the replicated plastic pipettes without the nanotexture at the tip.

Figure 6a shows the variation of contact angle as a function of the height of the nanostructure. For the flat surface, the contact angle of the nylon polymer is 74.7°. To increase the height of the nanostructure, the contact angle is decreased from 74° to 66°. The addition of the nanostructure changes the surface property to a more hydrophilic nature. According to the basic model with water droplet on the structure, the wetting property is determined by the position relationship between the water droplet and the nanostructure surface, based on the theory of the Cassie–Baxter model and the Wenzel model. In this case, the contact angle decreases slowly with increasing nanostructure height. Thus, we can conclude that the model that considers the water droplet on the surface nanostructure yields a balance between the Cassie–Baxter model and the Wenzel model, in terms of the trapped air. Therefore, when there is a large amount of water droplets (liquid water), the wetting model is shifted from the Cassie–Baxter model to the Wenzel model. The nanostructure surface is hydrophilic because water is inserted into the nanostructure by removing the trapped air. Figure 6b shows the measurement results of the wetting behaviour obtained by using the water-sprayed coating. For the flat surface, the wetting area ratio is 2.71%; the water droplet rarely showed minimal spreading on the plastic surface. In contrast, the wetting area ratio increases drastically from 20.2% to 92.6% with an increase in the height of the nanostructure. The water droplet on the plastic plate is completely covered by the increased nanostructure height. Thus, the combination of the nanostructure and the hydrophilic NY can enhance the hydrophilic property without additional coating or plasma treatment technology. Figure 7 shows photographs of the plastic capillary tube with and without the nanostructure.

**Figure 8:**
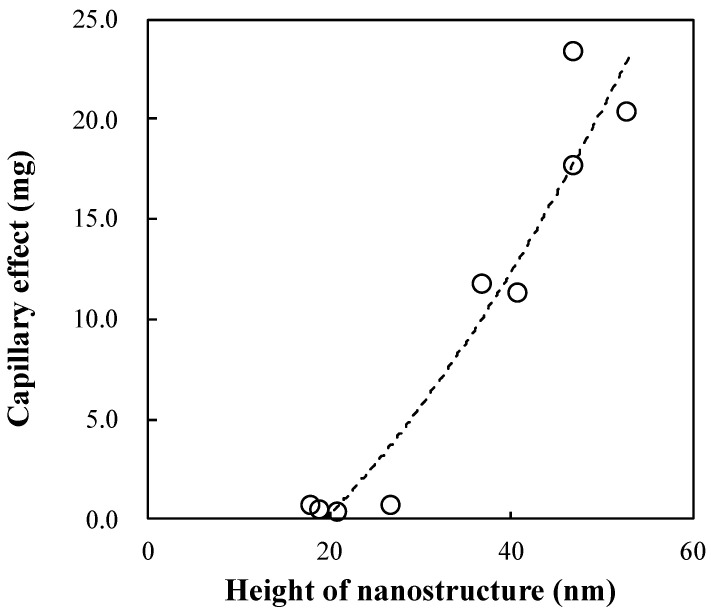
Relation between the capillary effect and the height of the nanotexture.

In the absence of a nanostructure, the capillary effect was not observed, owing to the hydrophobicity of the tip surface. The plastic capillary tube used in this study required additional coating or plasma treatment technology to produce the capillary effect. However, fabricating a nanostructure at the tip of the plastic tube enhanced the tube surface hydrophilicity, thereby producing the capillary effect in the plastic capillary tube. In addition, the plastic capillary tube exhibiting the capillary effect was fabricated by only a single replication process, which did not require additional coating or plasma treatment. Thus, using a plastic capillary tube with a nanostructure is a low-cost and simple process for decreasing contamination and enhancing surface wettability. Figure 8 shows the relationship between the capillary effect and the height of the nanostructure. The capillary effect increases linearly with increasing nanostructure height. Thus, although the nanostructure was grown only at the tip of the plastic capillary tube, it still produced the capillary effect in the tube. Consequently, an increase in the nanostructure height increased the capillary effect, which in turn enhanced the wettability.

Figure 2 also shows the capillary effect enhancement with changing wetting properties only at the tip of the capillary tube. Thus, we can conclude that the plastic tube with a nanostructure at its tip will be useful for biomedical applications. Additionally, we believe that the developed nanostructure fabrication method is effective for controlling the wetting properties of plastic surfaces and can enable the low-cost and high-throughput production of various components. Furthermore, the complex moulding technology for pipette replication with nanostructure has technological limitations. In addition, material selection limits the capillary action in a plastic tube. Therefore, we believe that the wetting enhancement technology, combined with plastic hydrophilicity, will be a more useful tool to improve the plastic surface wetting properties. In the future, our ultimate goal will be to find an effective way to enhance the capillary effect of a plastic tube by only injection moulding.

## 5. Conclusions

We investigated the capillary effect of a plastic capillary tube by using a nanostructure. In the calculation of the capillary effect using the plastic capillary tube, we found that the capillary effect was strongly dependent on the wetting property of the selected material. In addition, growing a nanostructure only at the tip of the plastic capillary tube increased the capillary effect by enhancing the hydrophilicity of the tube surface. Thus, we can conclude that there is a possibility of enhancing the capillary effect in a plastic capillary tube by increasing the hydrophilicity only at the tip of the tube. In the experimental results, NY or PMMA showed hydrophilic trends. In addition, by combining the nanostructure and the hydrophilic NY, the hydrophilicity was drastically increased by increasing the nanostructure height. The wetting area ratio was increased from 20.2% to 92.6% by increasing the height of the nanostructure from 40 nm to 100 nm. Thus, it was confirmed that the capillary effect can be enhanced by enhancing the hydrophilic property via a combination of the nanostructure and the hydrophilic NY. Furthermore, during the investigation of the capillary effect using the plastic capillary tube, the capillary effect surprisingly emerged in the plastic capillary tube when the nanostructure was included at the tip of the plastic tube. The capillary effect increased linearly with increasing nanostructure height. In addition, the plastic capillary tube exhibiting the capillary effect was fabricated by only a single replication process, which did not require additional coating or plasma treatment. Thus, we can conclude that a plastic capillary tube equipped with a nanostructure at its tip is highly effective for biomedical applications owing to its ability to reduce contamination in the human body. The proposed fabrication process is a low-cost and simple process for enhancing the wettability of the tube.

The simulations performed in this study were advantageous as a first step investigation of the fluid motions because it was easy to observe the interfacial motions near the solid surfaces and set the contact angles on the surfaces flexibly. Additionally, the computational costs for 2D simulations were much lower than those for 3D ones. In the future, the penetration of a two-phase fluid into an irregular capillary tube will be numerically simulated by extending the 2D model with a 3D spatial grid at a higher resolution. Such simulations can be used for the parametric study of the shape and surface wettability of a capillary tube, the physical properties of a fluid (e.g., interfacial tension and viscosity), etc. The effect of the surface nanostructure on the wettability will be considered in terms of variation in the contact angle, similar to the present study. Simultaneously, the micrometre-sized surface structures will be modelled approximately with cubic cells of a regular 3D spatial grid, similarly to the previous study on microdroplets on a grooved solid surface [34]. Moreover, fractal theory [39,40] will be applied to numerical simulations of fluid motion in a capillary tube—within the framework of macroscopic two-phase fluid dynamics—to study the effect of electrical charge on the nanostructured surface wettability and capillary pressure.

## Figures and Tables

**Table 1:** Water contact angle for several types of plastic materials.

Plastic Material	Water Contact Angle (deg)	Producer	Product Name
Nylon resin (NY)	74.7°	RIKEN TECHNOS Co. Ltd, Japan	Static Master: ESA-9166N
Polyacetal (POM)	76.2°	Polyplastics Co. Ltd, Japan	DURACON: 270-44
Acrylic Resin (PMMA)	79.7°	Mitsubishi Gas Chemical Company, Japan	Optimas: 7500
Cyclo olefin Copolymer (COC)	86.0°	Mitsui Chemicals, Japan	APEL: 5014DP
Acrylonitrile-Butadiene-Styrene Resin (ABS)	89.7°	Asahi Kasei Chemicals, Japan	Synthetic Resin: 191
Low-Density Polyethylene (LDPE)	91.8°	Nippon Polyethylene Co., Ltd, Japan	NOVATEK-LD:LB420M
Polystyrene Resin (PS)	95.5°	PS Japan	PSJ-Polystyrene:679
Polycarbonate Resin (PC)	95.9°	Mitsubishi Engineering Plastics, Japan	Eupilon: S 3000R
Cyclo olefin Polymer (COP)	96.1°	Nippon Zeon, Japan	Zonex:690R
Polybutyrene terephthalate Resin (PBT)	99.4°	Mitsubishi Engineering Co., Ltd, Japan	Novaduran: 5510S
Polypropylene Resin (PP)	101.2°	Nippon Polypro Co.,Ltd, Japan	NOVATEC-PP:MA 3H

## Nomenclature

*ρ_A_*, *ρ_B_*	Fluid phases
*μ_A_*, *μ_B_*	Viscosities: 1.0 × 10^−3^ Pa·s
*σ*	Interfacial tension between phases A and B: 7 × 10^−2^ N·m^−1^
*L*	Distance: 500 µm
*θ_W_,_in_*	Contact angles of the inner surface
*θ_W,out_*	Contact angles of the outer surface
*θ_W,1_*	Contact angle of the front faces of the plates
*U_in_*	Flows at a constant volumetric flow rate with a uniform velocity
*Oh*	Ohnesorge number: *Oh* = μ_A_/(ρ_A_σ*L*)^0.5^ = 5.35 × 10^−3^
*Re*	Reynolds number: *Re* = ρ_A_*LU_in_*/μ_A_ = 0.9982
*Ca*	Capillary number: *Ca* = μ_A_*U_in_*/σ = 2.857 × 10^−5^
*h*	Height of the liquid phase penetrating the vertical capillary tube
*σ*	Surface tension of the liquid phase
*r*	Radius of the capillary tube
*ρ*	Density of the liquid phase
*g*	Acceleration due to gravity
*∂s/L*	Interfacial displacement
